# Brominated Skeletal Components of the Marine Demosponges, *Aplysina cavernicola* and *Ianthella basta*: Analytical and Biochemical Investigations

**DOI:** 10.3390/md11041271

**Published:** 2013-04-17

**Authors:** Kurt Kunze, Hendrik Niemann, Susanne Ueberlein, Renate Schulze, Hermann Ehrlich, Eike Brunner, Peter Proksch, Karl-Heinz van Pée

**Affiliations:** 1 General Biochemistry, TU Dresden, Dresden 01062, Germany; E-Mails: kurt.kunze@chemie.tu-dresden.de (K.K.); Karl-Heinz.vanPee@chemie.tu-dresden.de (K.-H.P.); 2 Institute of Pharmaceutical Biology and Biotechnology, Heinrich Heine University Duesseldorf, Universitaetsstrasse 1, Geb. 26.23, Duesseldorf 40225, Germany; E-Mails: hendrik.niemann@uni-duesseldorf.de (H.N.); proksch@uni-duesseldorf.de (P.P.); 3 Bioanalytical Chemistry, TU Dresden, Dresden 01062, Germany; E-Mails: susanne.ueberlein@chemie.tu-dresden.de (S.U.); renate.schulze@chemie.tu-dresden.de (R.S.); 4 Institute of Experimental Physics, TU Bergakademie Freiberg, Freiberg 09596, Germany; E-Mail: hermann.ehrlich@physik.tu-freiberg.de

**Keywords:** demosponges, brominated tyrosine-derivatives, halogenase, HPLC, NMR, IR

## Abstract

Demosponges possess a skeleton made of a composite material with various organic constituents and/or siliceous spicules. Chitin is an integral part of the skeleton of different sponges of the order Verongida. Moreover, sponges of the order Verongida, such as *Aplysina cavernicola* or *Ianthella basta*, are well-known for the biosynthesis of brominated tyrosine derivates, characteristic bioactive natural products. It has been unknown so far whether these compounds are exclusively present in the cellular matrix or whether they may also be incorporated into the chitin-based skeletons. In the present study, we therefore examined the skeletons of *A. cavernicola* and *I. basta* with respect to the presence of bromotyrosine metabolites. The chitin-based-skeletons isolated from these sponges indeed contain significant amounts of brominated compounds, which are not easily extractable from the skeletons by common solvents, such as MeOH, as shown by HPLC analyses in combination with NMR and IR spectroscopic measurements. Quantitative potentiometric analyses confirm that the skeleton-associated bromine mainly withstands the MeOH-based extraction. This observation suggests that the respective, but yet unidentified, brominated compounds are strongly bound to the sponge skeletons, possibly by covalent bonding. Moreover, gene fragments of halogenases suggested to be responsible for the incorporation of bromine into organic molecules could be amplified from DNA isolated from sponge samples enriched for sponge-associated bacteria.

## 1. Introduction

Approximately 70% of the surface of our planet is covered by water, making the oceans the biggest habitat on earth that exhibits a high and still largely unexplored biodiversity. Numerous marine organisms are known to produce bioactive compounds [1,2], including, foremost, sponges [3]. Sponges are multicellular organisms [4,5] and represent the oldest and most primitive metazoans. These sessile animals have successfully adapted to various environments and show a global distribution. Sponges are not restricted to the seas, but occur also in fresh water, even though the majority of known sponge taxa live in the sea. In spite of their sessile nature and lack of morphological defense mechanisms (excluding spiculae), sponges, in most cases, successfully withstand predators, as well as overgrowth by fouling organisms. The evolutionary success of sponges is mainly due to an effective chemical defense that is based on deterrent, cytotoxic and/or antibiotically active compounds [3,6,7,8] that protect sponges from predators, such as fishes or mollusks, from overgrowth by fouling organisms and from infections caused by microbial pathogens.

Demosponges form the largest class of sponges [9]. They possess a skeleton made of a composite material with various organic constituents, such as proteins (spongin) [10], polysaccharides (chitin) [11,12] and/or siliceous spicules [9,13]. Chitin is an integral component of various invertebrates. Ehrlich *et al.* reported for the first time the presence of chitin as an integral part of the skeleton of different sponges of the order Verongida [11]. Meanwhile, chitin could also be found in the skeletons of further demosponges, like in species of the genus *Aplysina* [11,14], in *Verongula gigantea* [14] and in *Ianthella basta* [12]. All demosponge species, which were so far shown to exhibit a chitin-based skeleton, belong to the order Verongida.

**Figure 1:**
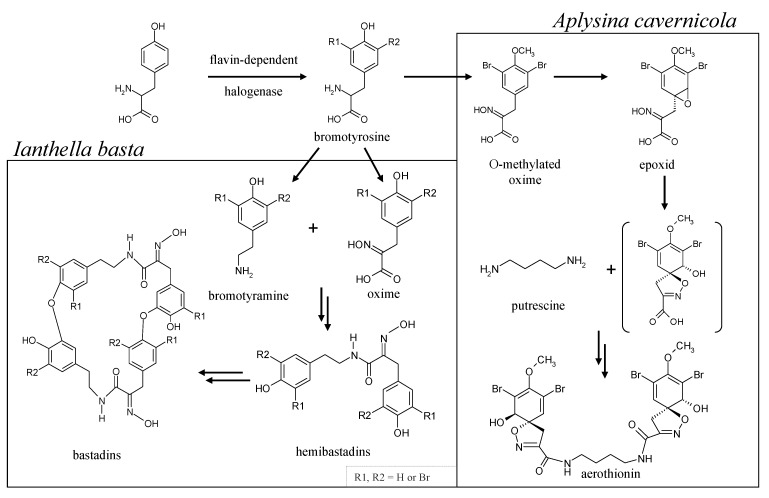
Hypothetical pathway for the biosynthesis of aerothionin in *A. cavernicola* and bastadins in *I. basta* according to Tymiak and Rinehart [21] and Leone-Stumpf [22]. Data available for biological halogenation reactions strongly suggest the involvement of flavin-dependent halogenases in the bromination reactions occurring during aerothionin and bastadin biosynthesis. The compound in brackets has not been isolated yet.

Moreover, the order Verongida is well-known for the biosynthesis of brominated tyrosine-derivates, such as bastadin derivates or isoxazoline alkaloids [15,16]. These secondary metabolites have been shown to act against predators [6], competing marine invertebrates [17] or against bacteria [15]. For example, brominated tyrosine-derived compounds from the Verongida sponge, *Aplysinella rhax*, moderately inhibit bacterial chitinase [18]. Earlier investigations [11,14] demonstrated that the chitin-based skeletons of Verongida sponges contain tightly attached/incorporated organic compounds of yet unknown structure. This leads to the question: which organic compounds in addition to chitin adhere to the skeletons? Since brominated compounds, such as bromotyrosine derivatives, are characteristic natural products of Verongida sponges, such as *Aplysina* sp. or *I. basta*, it is possible that these compounds are not only present in the cellular matrix, but may also be incorporated into the chitin-based skeletons. Early investigations showed the presence of aerothionin in the spherulous cells of the marine sponge *Aplysina fistularis* [19]. There is, however, also an earlier report demonstrating the localization of brominated compounds in the spherulous cells, as well as the skeletal fibers of *Aplysina aerophoba* [20]. That means the localization of bromotyrosine derivatives, as well as their origin within the sponge and the biosynthetic pathway leading to these molecules remain to be elucidated. Based on earlier studies [21,22], biosynthetic pathways leading to bastadins and isoxazoline alkaloids, such as aerothionin, can be proposed as described in Figure 1.

In recent years, it became increasingly evident that numerous compounds isolated from sponges are actually produced by bacterial symbionts [23]. The separation of the sponge from the bacterial symbionts with subsequent cultivation of the “bacterial free” sponge and the bacterial symbionts alone is not possible. Instead, the issue of the producing organism can be dealt with using molecular genetics. Accumulation of bacterial symbionts and isolation of their DNA allows the search for genes required for the biosynthesis of the respective metabolite using PCR primers or suitable probes for specific genes. In the case of bromotyrosines (aerothionin and bastadins; Figure 1) isolated from *A. cavernicola* and *I. basta*, suitable target genes are non-ribosomal peptide synthetase genes or genes coding for enzymes catalyzing the incorporation of bromine. In the last few years, it became evident that flavin-dependent halogenases are the type of halogenating enzymes involved in the regioselective incorporation of halogen atoms into aromatic and other compounds activated for attack by an electrophilic halogen species [24]. The data gathered on flavin-dependent halogenases using different substrates should allow the construction of specific probes and PCR primers for the search for halogenase genes involved in the biosynthesis of the bromotyrosine derivatives produced by *A. cavernicola* and *I. basta*. Cloning of the respective genes with larger flanking regions and subsequent sequencing should at least allow for the decision of whether the producing organism is a bacterial symbiont or a eukaryote. If the producing organism turns out to be a bacterial symbiont, cloning of the complete gene cluster will allow the identification of the genes required for the biosynthesis of these bromotyrosine derivatives and the elucidation of the biosynthetic pathway.

The goal of the present study was to examine the skeletons of *A. cavernicola* and *I. basta* with respect to bromotyrosine metabolites. If present, these compounds were to be identified and compared to those occurring in the cellular matrix. Additionally, the DNA of the sponges, *A. cavernicola* and *I. basta*, was to be analyzed for the presence of halogenase genes likely to be involved in the biosynthesis of the brominated sponge-derived alkaloids, thereby addressing the question of whether microbial symbionts are the true producers of these compounds. 

## 2. Results and Discussion

### 2.1. Isolation and Characterization of the Sponge Skeletons

**Figure 2:**
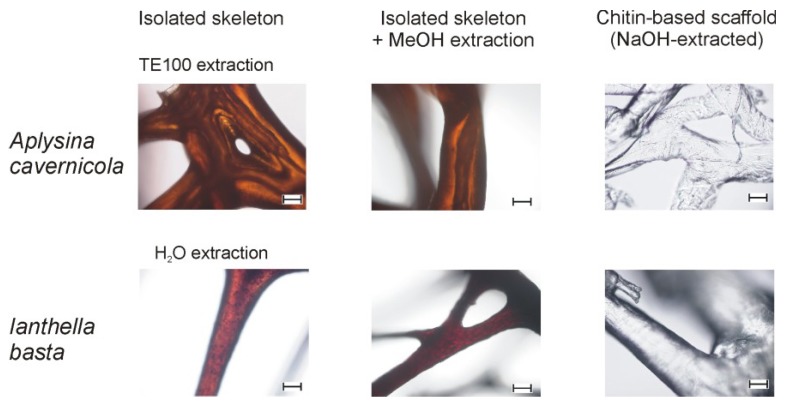
Scheme demonstrating the preparation of sponge skeletons, including skeleton isolation by H_2_O or TE100, MeOH-based extraction of the skeletons and extraction of the chitin-based scaffold by NaOH (top: *A. cavernicola*, scale bar: 100 µm; bottom: *I. basta*, scale bar: 100 µm). The treatment procedures are described in Section 3.

The formerly described isolation [14] of pure chitin-scaffolds from marine sponges—which are an integral part of the sponge skeletons—is based on treatment with NaOH. This alkaline extraction, however, results in the hydrolytic degradation of any other skeleton-associated or incorporated biomolecules, such as bromotyrosine derivatives. Therefore, an alternative, milder extraction method had to be developed, which allows effective extraction and cleaning of the intact skeletons from other sponge components without chemical deterioration of labile constituents. H_2_O and TE100 (10 mM Tris-HCl, 100 mM EDTA, pH 8) are “mild” solvents, which do not substantially modify biomolecules. Thus, TE buffers are commonly used in molecular biology/biochemistry to isolate, purify and store biomolecules (e.g., [25,26,27,28]). The optimized extraction procedure based on the treatment of the integer sponge with H_2_O and TE100 is described in the Experimental Section. Figure 2 displays the results of these extraction experiments. It could be shown that TE100 extraction is favorable, especially in the case of the skeletons of *A. cavernicola*, since H_2_O-based extraction took more than four weeks.

**Figure 3:**
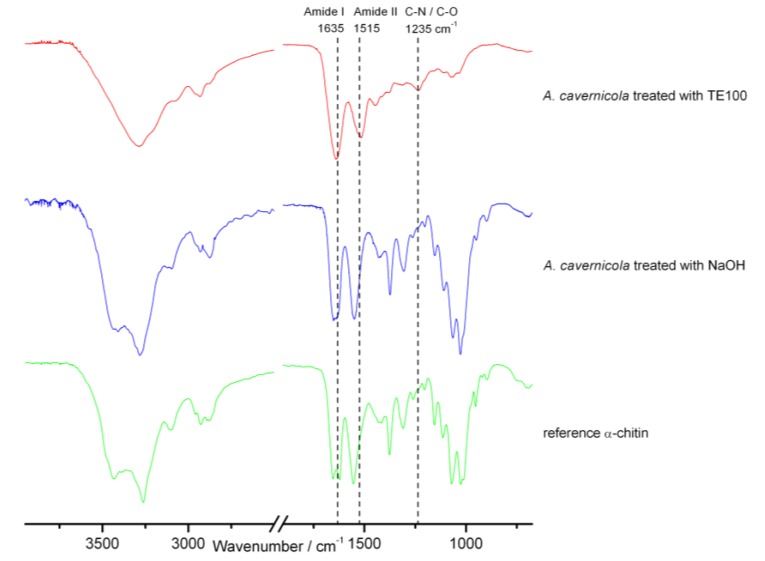
ATR FTIR spectra of the *A. cavernicola* skeleton samples after different isolation/preparation steps, except for MeOH extraction (see Figure 2). For comparison, the spectrum of an α-chitin reference sample is also shown. The assignment of the various bands is given in the Supporting Information, Table S1. The corresponding results obtained for *I. basta* are shown in the Supporting Information, Figure S1.

It was also observed that the skeleton of *I. basta* extracted with TE100 is slightly brighter compared to extraction with H_2_O. This indicates that pigments are removed from the skeletons of *I. basta* by TE100. H_2_O-based skeleton isolation was, therefore, preferred for *I. basta*. Apart from this observation, the microscopic images do not show any morphological damage caused by the extraction process. To analyze the chemical composition of the isolated skeletons, ATR FTIR and NMR studies were carried out. Figure 3 shows the ATR FTIR spectra of *A. cavernicola* after the different extraction steps before methanol extraction (see Figure 2). When comparing the spectra of the skeleton samples with those of the reference α-chitin, it becomes evident that characteristic vibrations of chitin are already visible in the TE100-treated sample. However, comparison of the spectra also reveals several striking differences. The amide II band occurs at 1550 cm^−1^ in pure chitin, whereas it is found at 1515 cm^−1^ in the skeleton samples. The intensity of both amide bands relative to the bands between 1000 and 1200 cm^−1^ (C–O stretching vibrations) is much higher in the skeleton samples compared with chitin. Moreover, the band at 1235 cm^−1^ observed for the skeleton samples does not occur in pure chitin. This wavenumber falls into the characteristic range of C–O and C–N stretching vibrations. These differences between the skeleton samples and pure chitin already indicate the presence of other organic compounds, probably amino acids/peptides/proteins or their derivatives attached to the chitin in the skeleton samples. Practically the same results were obtained for *I. basta* (see Supporting Information, Figure S1).

**Figure 4:**
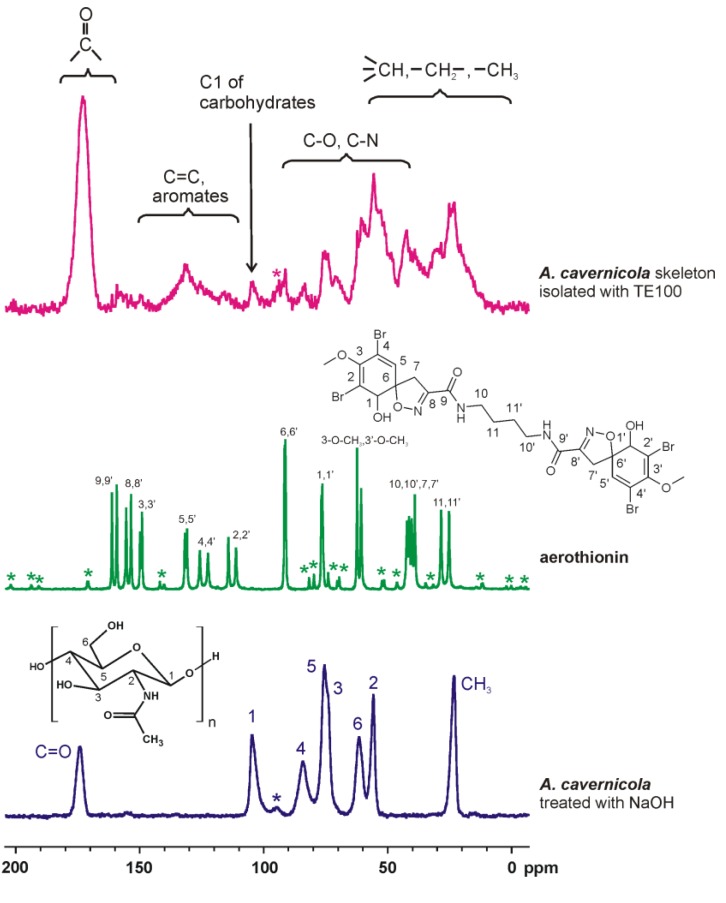
^13^C{^1^H} CP MAS NMR spectra of the skeletons of *A. cavernicola* after TE100 treatment. For comparison, the spectra of the pure chitin scaffold obtained after NaOH treatment and of pure aerothionin are also shown. The signal assignments follow from the inserts (for aerothionin, see, also, Supporting Information, Figure S2). The corresponding results obtained for *I. basta* are shown in the Supporting Information, Figures S3 and S4. * denotes spinning sidebands.

This is consistent with the observations made by ^13^C solid-state NMR spectroscopy. The ^13^C{^1^H} CP MAS NMR spectra of *A. cavernicola* (Figure 4) clearly show the presence of further organic components in addition to the chitin scaffold. The spectrum of aerothionin, a major bromotyrosine derivative found in *A. cavernicola*, is also shown in Figure 4. The ^13^C solid-state NMR spectrum of the *A. cavernicola* skeleton indeed shows several narrow signals characteristic of aerothionin, which are, however, very weak (see, also, Supporting Information, Figure S7). In addition to these narrow signals, the spectrum exhibits broader and more intense signals in the same characteristic regions of aromatic carbons/carbonyls, as well as in the aliphatic region, indicating the presence of organic material other than chitin in analogy to the infrared spectra described above. In order to prove that bromotyrosines or their derivatives may be present in the isolated sponge skeletons, the bromine content of the skeleton samples was measured by potentiometric titration following the protocol described in Section 3. (see also [29]). A bromine content of 40 µg Br per milligram sponge skeleton was measured before MeOH treatment (see below, Table 1).

**Table 1:** Bromine contents of *A. cavernicola* and *I. basta* after different isolation/extraction steps measured by potentiometry. * Note that the bromine concentrations are related to the dry weight of the respective samples after the different isolation/extraction steps, thus representing the relative bromine contents in the remaining material.

	*A. cavernicola* mg Br/g dry weight *	*I. basta* mg Br/g dry weight *
Sponge tissue	60 ± 5	72 ± 10
Skeleton (isolated)	40 ± 3	51 ± 4
Skeleton after MeOH extraction	35 ± 2	44 ± 4
Scaffold after NaOH treatment	0	0

In summary, it can, therefore, be stated that bromotyrosines or bromotyrosine derivatives may indeed be tightly bound to the skeletons. Since the extraction of these compounds from integer sponge samples usually relies on a MeOH-based extraction procedure [30], we have chosen this established protocol in order to extract the—spectroscopically detected—skeleton-associated compounds.

### 2.2. The Effect of MeOH Extraction upon the Isolated Sponge Skeletons

Both the MeOH-treated sponge skeletons and the MeOH extracts obtained from *A. cavernicola* and *I. basta* skeleton samples were analyzed. Surprisingly, the MeOH treatment did not result in any pronounced changes of the ATR FTIR and ^13^C{^1^H} CP MAS NMR spectra compared with the skeletons before MeOH extraction (see Supporting Information, Figures S5 and S6). This indicates that the established MeOH-based method extracts only small amounts of organic material from the skeletons. Most importantly, in order to decide whether or not brominated organic compounds are found among the remaining MeOH-insoluble, *i.e.*, tightly skeleton-associated organic material, the bromine content in the samples after the different isolation/extraction steps was measured by potentiometry. The results of these measurements are summarized in Table 1. Both sponges exhibit a similar behavior. The sponge tissue before any extraction procedure exhibits the highest relative amount of bromine. The isolated skeleton samples possess lower bromine concentrations. That means that the soluble parts of the sponge—which are removed from the skeletons by TE100 or H_2_O treatment—contain higher concentrations of brominated compounds than the skeletons. Nevertheless, the isolated skeleton samples do still exhibit a significant amount of bromine-containing compounds, which are obviously not soluble in TE100 or H_2_O. Subsequent MeOH treatment only results in the removal of a rather insignificant amount of bromine from the skeletons for both sponges, indicating that the majority of brominated skeletal compounds is strongly—maybe covalently—bound to the skeletons. In contrast, the NaOH treatment results in the complete removal of bromine from the sponge skeletons. These observations are in agreement with the described spectroscopic observations showing that the strongly skeleton-associated organic compounds are completely removed from the chitin-based scaffolds by NaOH. That means that the bromine found in the sponge skeletons is mainly associated with the NaOH-extractable, strongly skeleton-associated organic material.

**Figure 5:**
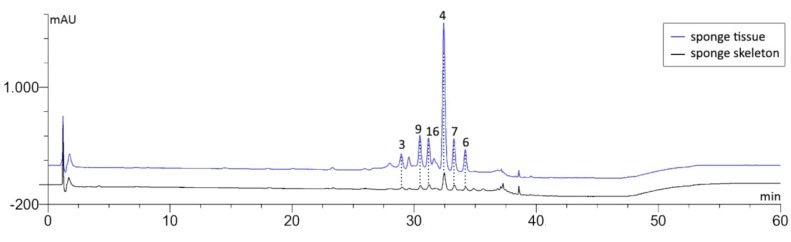
HPLC-DAD chromatograms of fresh sponge and H_2_O-treated skeleton extracts of *I. basta* with the peaks of identified bastadins 3, 4, 6, 7, 9 and 16 detected at λ = 235 nm.

Moreover, the MeOH extracts were analyzed by the established HPLC-based method (see Experimental Section). HPLC analysis of a skeleton extract from *A. cavernicola* showed trace amounts of aerothionin, which was confirmed by the on-line recorded UV spectrum and comparison of the retention time with an authentic standard. The concentration was, however, too small to allow quantification. Several compounds with UV spectra typical for bromotyrosine derivatives were detected by HPLC in the MeOH extracts of *I. basta*. Comparison of the HPLC profiles from the skeleton extracts obtained from H_2_O-treatment with a crude MeOH extract of fresh sponge tissue from the same specimen of *I. basta* (Figure 5) shows a high degree of analogy.

Comparison of the retention times of the six major peaks in the chromatogram of the H_2_O-treated skeleton extract with authentic standards isolated from the same sponge specimen indicates the presence of bastadins 3, 4, 6, 7, 9 and 16 (see Supporting Information, Figure S8, for structures). Further confirmation for this tentative identification was achieved by LC-MS and comparison of the resulting pseudomolecular ions. A typical molecular ion cluster of a brominated compound was found in the negative mode at *m/z* 941.1 [M − H]^−^, which indicates the presence of bastadin 3 (see Supporting Information, Figure S9, for the spectrum). HPLC-based comparison of the bastadin profiles present in extracts of sponge tissue and in extracts of the skeleton revealed the same major constituent (bastadin 4, Table 2) for both extracts, albeit at different percentages. Whereas bastadin 4 accounted for 56% of all detected bastadin derivatives in the tissue extract, this number decreased to 41% in the extract obtained from the skeleton. The percentages of several minor bastadins showed an opposite trend. For example, whereas bastadin 16 amounts to 10% of the bastadin profile from sponge tissue, the same compound reaches 20% in extracts from the skeleton. However, the total bastadin concentration found in the skeleton extracts of *I. basta* amounts to only 0.51 mg per gram dry weight. In contrast, an almost 300-fold higher total bastadin concentration was measured for the tissue extract (Table 2). This shows that most of the previously identified MeOH-extractable bromotyrosine derivatives, such as aerothionin and bastadins, were already removed from the sponge skeletons by TE100 or H_2_O, indicating the localization of these compounds in sponge cells and/or symbionts.

In summary, it can therefore be stated that the total concentration of MeOH-soluble bromotyrosine derivatives in the sponge skeletons is very low for both species under study. It is, however, remarkable that the sponge skeletons contain a considerable amount of bromine even after exhaustive MeOH extraction. It should be noted that these compounds, which are tightly bound to the skeleton, could not be extracted by other common solvents, such as acetone, urea and ethylenediaminetetraacetic acid/sodium dodecyl sulfate. Therefore, we conclude that the skeletons contain insoluble, *i.e.*, strongly bound brominated compounds, which have not yet been identified.

**Table 2:** Contents of known bastadins identified in MeOH extracts of the *I. basta* skeleton and in sponge tissue.

Bastadin No.	Bastadin Content in Isolated Skeleton/mg g^−1^ dry weight	Bastadin Content in Sponge Tissue/mg g^−1^ dry weight
3	0.02	7
9	0.05	15
16	0.10	14
4	0.21	77
7	0.07	15
6	0.06	10
total	0.51	138

### 2.3. Genetic Analyses

**Figure 6:**
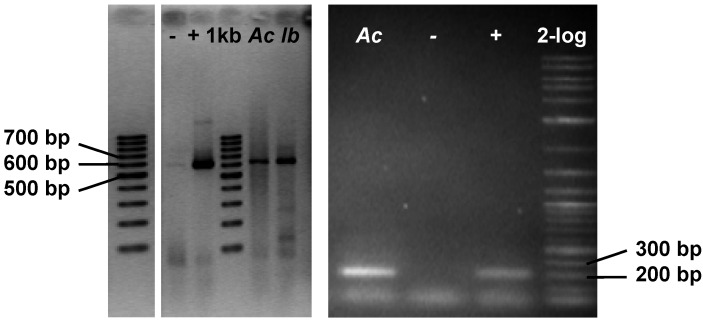
Detection of genes of flavin-dependent halogenases in metagenomic DNA of *A. cavernicola* (*Ac*) and *I. basta* (*Ib*) via degenerated primer PCR; left: primer pair TyrhalA_for/rev; right: primer pair HaloDetect_for/rev; +: fragment of *clohal* (flavin-dependent halogenase from the clorobiocin biosynthetic gene cluster) as a positive control; −: H_2_O as a negative control.

**Figure 7:**
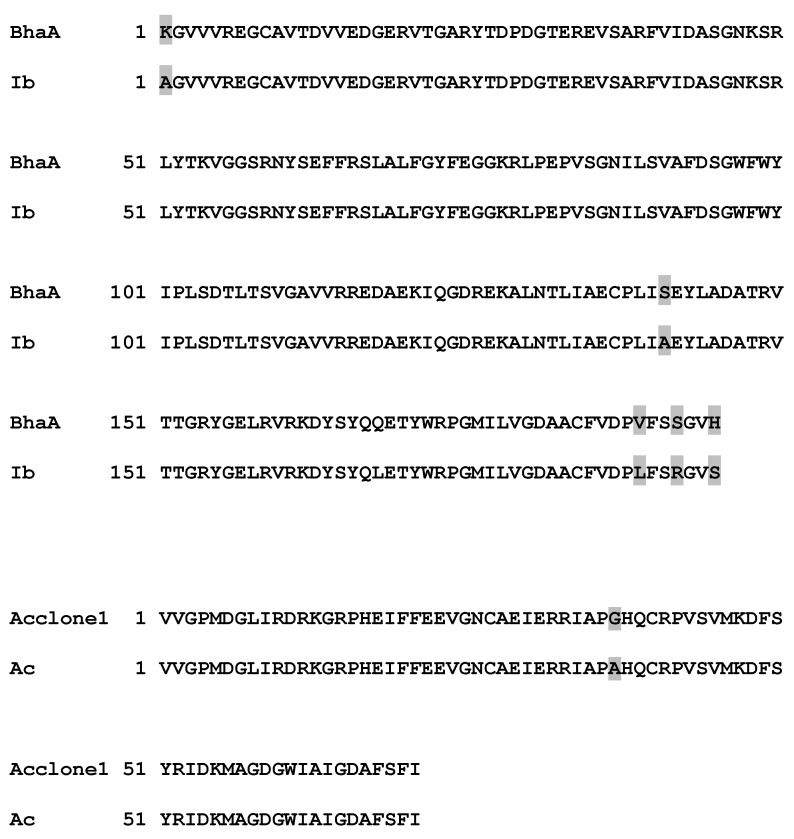
Comparison of the amino acid sequence derived from the 600 bp halogenase gene fragment obtained from bacteria associated with *I. basta* (Ib) to the halogenase BhaA from balhimycin biosynthesis [31] and of the 300 bp halogenase gene fragment from bacteria associated with *A. cavernicola* (Ac) with a halogenase gene fragment (Acclone 1) isolated by Bayer *et al.* from *A. cavernicola* [23]. Non-identical amino acids are shaded in grey.

DNA isolated from samples of the two sponges enriched for bacterial symbionts was found to be contaminated with pigments and required further purification to allow for its use in PCR and cloning experiments. This purification was achieved by size exclusion chromatography (see Supporting Information, Figure S10). Using the purified DNA, 600 bp gene fragments could be detected by PCR (Figure 6). The fragment obtained from bacteria associated with *I. basta* showed high homology to known genes of flavin-dependent halogenases accepting tyrosine or tyrosine derivatives as substrates. The amplified fragment from symbionts of *I. basta* shows high similarity (98%) to *bhaA*, a flavin-dependent halogenase from balhimycin biosynthesis in the bacterium, *Amycolatopsis balhimycina* (Figure 7, [31]). Like in bastadins and aerothionin, balhimycin contains a halogenated tyrosine derivative, indicating that the amplified halogenase gene fragment might also code for a halogenase accepting tyrosine or a tyrosine derivative as a substrate and might thus be part of the bastadin biosynthetic gene cluster. The high similarity to *bhaA* also suggests that the genes for bastadin biosynthesis originate from symbiotic bacteria. Hence, these genes should be clustered, too. The 600 bp gene fragment obtained from the bacterial symbionts enriched from *A. cavernicola* could not be sequenced. Thus, a second primer pair (HaloDetect_for/rev) was used, which allowed the PCR-amplification of a 300 bp gene fragment (Figure 6). The amino acid sequence derived from this gene fragment is almost identical (99%) to a putative halogenase gene fragment cloned from *A. cavernicola*-associated bacteria [23]. However, the substrate of this halogenase, as well as the putative biosynthetic pathway it might be involved in, are yet unknown. In order to obtain information about the regions flanking the halogenase genes and to allow cloning of the biosynthetic gene clusters, metagenomic cosmid libraries will be constructed and screened for the halogenase genes using the amplified fragments as probes. It can, however, be stated that our genetic analyses point towards the presence of halogenase genes of symbiotic origin. This is in line with the above described observation that the majority of the previously known bromotyrosine derivatives can be removed from the sponge skeletons simply by TE100 or H_2_O. The above-described observation of strongly skeleton-associated brominated compounds raises the question of whether or not these substances are also of symbiotic origin.

## 3. Experimental Section

### 3.1. Isolation of the Skeletons

#### 3.1.1. Sponge Samples

The *A. cavernicola* sample was collected in the Mediterranean Sea (Hydra-Institute, Elba, Italy, www.hydra-institute.com [32]) and purchased from the Hydra Institut für Meereswissenschaften AG (Munich, Germany). The entire sponge was shock frozen immediately after underwater collection and was always kept frozen on dry ice during transport and storage. *I. basta* samples were collected by Prof. P. Schupp at Western Shoals in Apra Harbor (Guam); see also [12].

#### 3.1.2. H_2_O Extraction

Small pieces of *A. cavernicola* (approximately 10 g wet sponge) or *I. basta* (5–10 cm²) were soaked in 40 mL of distilled water for two weeks. Subsequently, the samples were transferred into freshly distilled water for 24 h. This procedure was repeated two times under continuous shaking.

#### 3.1.3. TE100 Extraction

Small pieces of *A. cavernicola* (approximately 10 g wet sponge) or *I. basta* (5–10 cm^2^) were soaked in 40 mL of TE100 (10 mM Tris-HCl, 100 mM EDTA, pH 8) for two weeks. Subsequently, the samples were transferred into freshly distilled water for 24 h. This was repeated six times under continuous shaking.

#### 3.1.4. NaOH Extraction

Alkaline extraction was performed for extraction of the pure chitin-based skeletons, which were used as a reference. According to [14], the samples were treated with 2.5 M NaOH for 24 h. The remaining fibrous skeletal material was neutralized. In a second step, the samples were treated with 20% acetic acid for 24 h. Subsequently, the remaining fibrous skeleton material was neutralized. This procedure was repeated until a colorless fibrous material remained.

#### 3.1.5. Methanol Extraction of the Purified Skeletons

The purified skeletons were put into 200 mL of methanol and sonicated for 1 min. Under stirring, the samples were treated with methanol for 24 h. Subsequently, the solvent was changed, and the skeletons were stirred in fresh methanol for a further 24 h. This procedure was repeated two times. Finally, all methanol extracts were combined, reduced in volume with a rotary evaporator and analyzed by HPLC. For *A. cavernicola*, extraction with methanol was followed by an acetonitrile extraction. For this purpose, the skeletons were put into 200 mL of acetonitrile and stirred for 24 h. Subsequently, the solvent was changed, and the skeletons were stirred in fresh acetonitrile for further 24 h. All acetonitrile extracts were combined, reduced in volume and analyzed by HPLC. Finally, the extracted skeletons were freeze-dried.

### 3.2. HPLC

#### 3.2.1. General Procedures

All analytical HPLC-DAD measurements were carried out on a Dionex Ultimate 3000 System employing a Knauer VertexPlus Column (125 × 4 mm, Eurospher 100–10, C18). The different gradient settings for analysis of samples from both sponges are shown in the Supporting Information, Table S2. The flow rate was adjusted to 1 mL min^−1^. The detection wavelength was set to λ = 235 nm. Crude extracts of *A. cavernicola* and of *I. basta* skeletons were dissolved in MeOH prior to HPLC analysis. Low resolution ESI mass spectra were recorded on-line using a Thermoquest Finnigan LCQDeca connected to an Agilent 1100 Series LC.

#### 3.2.2. Identification of Brominated Metabolites

Brominated metabolites present in the skeleton extracts were detected based on their retention times, a comparison with authentic standards and on their on-line ESI mass spectra.

#### 3.2.3. Quantification of Brominated Metabolites

The identified metabolites were quantified by peak integration using an external standard. Bastadin 3 was utilized as the external standard for bastadin derivatives from *I. basta* to establish the calibration graph shown in the Supporting Information (Figure S11). The contents of bromotyrosine metabolites were calculated as mg g^−1^ of the dried skeleton or of freeze dried sponge tissue.

### 3.3. Light Microscopy

Small pieces of the skeletons were put on a sample holder. Microscopic studies were carried out on a Keyence BZ-8000K microscope. The images were acquired at 10-fold magnification.

### 3.4. FTIR Spectroscopy

IR spectra were recorded using a Bruker FTIR spectrometer IFS 88. The samples were deposited on SPECAC Golden-Gate-ATR equipment. The spectra were measured in the range from 4000 cm^−1^ to 650 cm^−1^ with a spectral resolution of 0.5 cm^−1^. Each spectrum was recorded by the accumulation of 1000 scans. Subsequently, an ATR intensity correction was carried out. The spectra were baseline corrected and normalized to the most intensive band at about 1625 cm^−1^.

### 3.5. NMR Spectroscopy

Solid-state ^13^C NMR spectra of the skeletons of the sponges were recorded on a Bruker Avance 800 spectrometer at a ^13^C resonance frequency of 201.19 MHz using a commercial 3.2 mm triple-resonance (^1^H, ^13^C, ^15^N) E-free MAS NMR probe. During signal acquisition, SPINAL ^1^H-decoupling was applied [33]. The MAS frequency was 16 kHz. The spectra were recorded with a recycle delay of 3 s.

### 3.6. Bromine Determination by Potentiometric Titration

Small pieces (approximately 10 mg dry sample) of the samples were combusted by the method of Schöniger [29]. The samples were wrapped in ashless filter paper and trapped in a platinum grid, which was attached to the stopper of an Erlenmeyer flask. The Erlenmeyer flask contained 10 mL ultrapure water acidified with 2% HNO_3_ as the absorbent. The flask was flushed with pure oxygen, while the sample was ignited and put in the Erlenmeyer flask to ensure complete burning without loss. For complete absorption, the flask was allowed to equilibrate overnight. Afterwards, 2 mL of the resulting solution were diluted to 20 mL and measured by potentiometric titration. Accordingly, a silver nitrate solution (2 mM for whole sponge, 0.5 mM for skeletons) was added in 0.1 mL increments (see Supporting Information, Figure S12). To validate the described sample combustion, the amount of bromine in a reference sample (4-bromochlorobenzene) of known bromine concentration was measured. Within a recovery rate of 95% (±10%), the expected bromine concentrations could be detected by the described combustion and potentiometric titration method. In addition, the accuracy of this method was tested by adding 1 mL of a 1 mM KBr-solution to several digested samples. The corresponding amount of added bromine could also be detected within the experimental error of 10%, demonstrating the validity of the applied method.

### 3.7. DNA Analysis

#### 3.7.1. Enrichment of Symbiotic Bacteria

The symbiont enrichment procedure was adapted from Ouyang *et al*. [28]. Two grams of sponge material were sliced into small pieces and homogenized using a mortar and a pestle. Cells were suspended in 10 mL TE buffer (10 mM Tris-HCl, 1 mM EDTA, pH 8.0, 4 °C) and filtered through a nylon mesh (42 µm pore size). To eliminate sponge debris and dirt, the suspension was centrifuged at 250× *g* for 1 min at 4 °C. Cells were pelleted at 8000× *g* for 20 min at 4 °C. Finally, the cell pellet was washed three times with 10 mL TE100 buffer (10 mM Tris-HCl, 100 mM EDTA, pH 8.0, 4 °C) to remove sponge pigments. Microscopic analysis of the obtained cells revealed that the samples still contained sponge cells and a comparably high amount of bacterial cells.

#### 3.7.2. Extraction of Metagenomic DNA

According to Schirmer *et al.* [34], 0.5 g of symbiont enriched cell pellets were resuspended in 2 mL lysis buffer (0.5 M NaCl, 10 mM Tris-HCl, 100 mM EDTA, pH 8.0) and treated with lysozyme (200 mg mL^−1^ for 1 h at 37 °C), followed by a second lysis step with 1% SDS and 0.5 mg mL^−1^ proteinase K for 2 h at 50 °C. The DNA was purified three times by phenol-extraction, followed by one chloroform extraction step and finally precipitated over night at −20 °C with isopropanol. The DNA pellet was washed with ice cold 70% ethanol and dissolved in 100 µL H_2_O.

#### 3.7.3. Purification of DNA by Size Exclusion Chromatography

The solution of the extracted DNA still showed brownish contaminations (probably humic/fulvic acid-like substances), which inhibited further applications, such as digestion with restriction enzymes and PCR. To eliminate these contaminations, a size exclusion chromatographic step using a Superdex 200 column (Pharmacia Biotech) was employed. The molecular weight of humic/fulvic acid-like contamination is much smaller than 200 kDa. DNA with the size of 10 kb has an approximate molecular mass of 600 MDa. DNA after extraction had an average length between 20 and 40 kb. Thus, pure DNA will be in the last fractions of the void volume, whereas contaminations will elute much later. 1 mL of DNA solution in TEN buffer (10 mM Tris-HCl, 1 mM EDTA, 150 mM NaCl, pH 8.0) was loaded onto the column and eluted with TEN buffer. Elution was monitored at 254 nm, and fractions containing the high molecular weight DNA were collected and pooled. To avoid digestion of the DNA by contaminating nucleases, a final phenol extraction step was used. The purified DNA was diluted in ddH_2_O.

#### 3.7.4. Detection of Halogenase Genes

The degenerated primer pair (TyrhalA_for: 5′-TACCAGGTCGAGCGSDBNMVNTCCGAC-3′ and TyrhalA_rev: 5′-CGGGACSACGAARCASGCSGCGTCBCC-3′) and (HaloDetect_for: 5′-GGACGGCTGGTTCTGGNHNATHCC-3′ and HaloDetect_rev: 5′-CACGCCGCGGGAGWANANNGG RTC-3′) were constructed using sequence data of published and unpublished data for identified and potential tyrosine halogenases. These primers were used for PCR for the detection of halogenase genes. Gene fragments were amplified with *taq*-polymerase (Fermentas) in ThermoPolII-buffer (NEB) containing 2 nmol MgCl_2_, 4% DMSO, 1 pmol of the degenerated primer pair and 0.1 pmol dNTPs in a total volume of 25 µL. One microliter of metagenomic DNA (approx. 20 ng µL^−1^) was used as the template. Initial denaturation was performed at 98 °C for 5 min. Elongation was carried out at 68 °C for 1 min (600 bp fragment) and 40 s (250 bp fragment) after a 30 s annealing period at 55 °C. Denaturation between cycles was done at 98 °C for 30 s. The final elongation step was performed at 68 °C for 5 min. PCR fragments obtained after 30 cycles were isolated using the GeneJET™ Purification Kit (Fermentas) and sequenced (MWG-eurofins). A BLAST search using the amino acid sequences derived from the DNA sequences of the fragments was performed to detect similarities with published halogenases.

## 4. Conclusions

In summary, the following conclusions can be drawn from our study:

(i) Genetic analyses reveal the presence of flavin-dependent halogenase genes in the sponges, *A. cavernicola* and *I. basta*. These genes are likely to originate from symbionts of the sponges. This agrees well with the observations of Bayer * et al.* [23]. Both gene fragments show high similarity to bacterial halogenase genes. It can, therefore, be assumed that the previously identified bromotyrosine derivatives in *A. cavernicola* and *I. basta*—aerothionin and bastadins—respectively, are probably of symbiotic origin.
(ii) Moreover, our analytical studies show that the majority of the previously identified bromotyrosine derivatives are not associated with the sponge skeletons. They can easily be removed from the skeletons by TE100 or even H_2_O treatment. This is in line with the aforementioned conclusion that these compounds are produced by symbionts.
(iii) However, a considerable amount of bromine-bearing organic molecules were found to be MeOH-insoluble. These strongly skeleton-associated compounds withstand the established extraction protocol and remain tightly associated with the sponge skeleton. It is tempting to speculate that these compounds are involved in the chemical defense of the skeleton, e.g., by inhibiting chitinases as discussed in the introduction section or by other biological activities. The extraction of these yet unidentified molecules—which may even be covalently bound to the chitin-based scaffolds—without severe chemical damage remains to be the subject of future work, including the elucidation of their ecological function/biological activity and biosynthetic pathway.

## Supplementary Files

Supplementary File 1 Supporting Information (PDF, 1009 KB)

## References

[1] Blunt J.W., Copp B.R., Hu W.P., Munro M.H.G., Northcote P.T., Prinsep M.R. (2009). Marine natural products. Nat. Prod. Rep..

[2] Montaser R., Luesch H. (2011). Marine natural products: A new wave of drugs?. Future Med. Chem..

[3] Proksch P., Putz A., Ortlepp S., Kjer J., Bayer M. (2010). Bioactive natural products from marine sponges and fungal endophytes. Phytochem. Rev..

[4] Müller W.E.G. (1998). Origin of metazoa: Sponges as living fossils. Naturwissenschaften.

[5] Li C.-W., Chen J.-Y., Hua T.-E. (1998). Precambrian sponges with cellular structures. Science.

[6] Thoms C., Wolff M., Padmakumar K., Ebel R., Proksch P. (2004). Chemical defense of Mediterranean sponges *Aplysina cavernicola* and *Aplysina aerophoba*. Z. Naturforsch. C.

[7] Thoms C., Ebel R., Proksch P. (2006). Activated chemical defense in *Aplysina* sponges revisited. J. Chem. Ecol..

[8] Paul V.J., Ritson-Williams R., Sharp K. (2011). Marine chemical ecology in benthic environments. Nat. Prod. Rep..

[9] Wehner R., Gehring W.J. (2007). Zoologie.

[10] Bergquist P.R., Cook S.D.C., Hooper J.N.A., van Soest R.W.M. (2002). Order Verongida Bergquist, 1978. Systema Porifera: A Guide to the Classification of Sponges.

[11] Ehrlich H., Maldonado M., Spindler K.-D., Eckert C., Hanke T., Born R., Goebel C., Simon P., Heinemann S., Worch H.  (2007). First evidence of chitin as a component of the skeletal fibers of marine sponges. Part I. Verongidae (Demospongia: Porifera). J. Exp. Zool. B Mol. Dev. Evol..

[12] Brunner E., Ehrlich H., Schupp P., Hedrich R., Hunoldt S., Kammer M., Machill S., Paasch S., Bazhenov V.V., Kurek D.V. (2009). Chitin-Based scaffolds are an integral part of the skeleton of the marine demosponge *Ianthella basta*. J. Struct. Biol..

[13] Uriz M.-J., Turon X., Becerro M.A., Agell G. (2003). Siliceous spicules and skeleton frameworks in sponges: Origin, diversity, ultrastructural patterns, and biological functions. Microsc. Res. Tech..

[14] Ehrlich H., Ilan M., Maldonado M., Muricy G., Bavestrello G., Kljajic Z., Carballo J.L., Shiaparelli S., Ereskovsky A.V., Schupp P. (2010). Three-Dimensional chitin-based scaffolds from Verongida sponges (Demospongiae: Porifera). Part I. Isolation and identification of chitin. Int. J. Biol. Macromol..

[15] Teeyapant R., Woerdenbag H., Kreis P., Hacker J., Wray V., Witte L., Proksch P. (1993). Antibiotic and cytotoxic activity of brominated compounds from the marine sponge *Verongia aerophoba*. Z. Naturforsch. C.

[16] Faulkner D.J. (2000). Marine pharmacology. Antonie Van Leeuwenhoek.

[17] Weiss B., Ebel R., Elbrächter M., Kirchner M., Proksch P. (1996). Defence metabolites from the marine sponge *Verongia aerophoba*. Biochem. Syst. Ecol..

[18] Tabudravu J.N., Eijsink V.G.H., Gooday G.W., Jaspars M., Komander D., Legg M., Synstad B., van Aalten D.M.F. (2002). Psammaplin A, a Chitinase Inhibitor Isolated from the Fijian Marine Sponge *Aplysinella rhax*. Bioorg. Med. Chem..

[19] Thomson J.E., Barrow K.D., Faulkner D.J. (1981). Localization of two brominated metabolites, aerothionin and homoaerothionin, in spherulous cells of the marine sponge *Aplysina fistularis* (=*Verongia thiona*). Acta Zool..

[20] Turon X., Becerro M.A., Uriz M.J. (2000). Distribution of brominated compounds within the sponge *Aplysina aerophoba*: Coupling X-ray microanalysis with cryofixation techniques. Cell Tissue Res..

[21] Tymiak A.A., Rinehart L.R. (1981). Biosynthesis of dibromotyrosine-derived antimicrobial compounds by the marine sponge *Aplysina fistularis* (*Verongia aurea*). J. Am. Chem. Soc..

[22] Leone-Stumpf D.  (2011). Synthesis and Chromatography of [RuCp]^+^-labelled Diaryl Ether Peptoids as Precursors of the Bastadins from the Marine Sponge *Ianthella basta*. PhD Thesis.

[23] Bayer K., Scheuermayer M., Fieseler L., Hentschel U. (2013). Genomic mining for novel FADH_2_-dependent halogenases in marine sponge-associated microbial consortia. Mar. Biotechnol..

[24] Van Pée K.-H. (2012). Enzymatic chlorination and bromination. Methods Enzymol..

[25] Chow T.Y.K. (1989). Purification of yeast—*E. coli* shuttle plasmid suitable for high transformation frequency in *E. coli*. Nucleic Acids Res..

[26] Bollet C., Gevaudan M.J., de Lamballerie X., Zandotti C., de Micco P. (1991). A simple method for the isolation of chromosomal DNA from Gram positive or acid-fast bacteria. Nucleic Acids Res..

[27] Wang H., Zhang L., Zhang F., An H., Chen S., Li H., Wang P., Wang X., Wang Y., Yang H. (2007). Investigation on the morphology of precipitated chemicals from TE buffer on solid substrates. Surf. Rev. Lett..

[28] Ouyang Y., Dai S., Xie L., Ravi Kumar M.S., Sun W., Sun H., Tang D., Li X. (2010). Isolation of high molecular weight DNA from marine sponge bacteria for BAC library construction. Mar. Biotechnol..

[29] Schöniger W. (1995). Eine mikroanalytische Schnellbestimmung von Halogenen in organischen Substanzen. Mikrochem. Acta.

[30] Ebada S.S., Edrada R.A., Lin W., Proksch P. (2008). Methods of isolation, purification and structural elucidation of bioactive secondary metabolites from marine invertebrates. Nat. Protoc..

[31] Pelzer S., Süßmuth R., Heckmann D., Recktenwald J., Huber P., Jung G., Wohlleben W. (1999). Identification and analysis of the balhimycin biosynthetic gene cluster and its use for manipulating glycopeptide biosynthesis in *Amycalotopsis mediterranei* DSM5908. Antimicrob. Agents Chemother..

[32] Institut für angewandte Hydrobiologie; HYDRA AG; HYDRA Institut für Meereswissenschaften AG; HYDRA Büro für Gewässerökologie Mürle & Ortlepp GbR; HYDRA Wiesloch—Dipl-Biol. Andreas Becker. http://www.hydra-institute.com.

[33] Fung B.M., Khitrin A.K., Ermolaev J. (2002). An improved broadband decoupling sequence for liquid crystals and solids. J. Magn. Reson..

[34] Schirmer A., Gadkari R., Reeves C.D., Ibrahim F., DeLong E.F., Hutchinson C.R. (2005). Metagenomic analysis reveals diverse polyketide synthase gene clusters in microorganisms associated with the marine sponge *Discodermia dissoluta*. Appl. Environ. Microbiol..

