# Supplementary material for: Brominated Skeletal Components of the Marine Demosponges, Aplysina cavernicola and Ianthella basta: Analytical and Biochemical Investigations
Source: Mar Drugs. 2013 Apr 17;11(4):1271–87. doi: 10.3390/md11041271 (PMC3705403; doi:10.3390/md11041271)
Supplement: Supplementary File 1 — Supporting Information (PDF, 1009 KB) [file marinedrugs-11-01271-s001.pdf]

Supporting Information

Table S1. Assignment of the IR bands in Figure 3 and Figure S1 (below).

A. cavernicola **(H_2O/TE100)**		***A. cavernicola*** **(NaOH)**	***I. basta*** **(H_2O/TE100)**		***I. basta*** **(NaOH)**	**α-chitin**	**Assignment**
Wavenumber/ cm^{-1}		Wavenumber/ cm^{-1}	Wavenumber/ cm^{-1}		Wavenumber/ cm^{-1}	Wavenumber/ cm^{-1}	
		898			897	898	CH_x deformation (o.o.p.)
				921		920	
		949			950	953	
1033		1027	1037		1029	1025	C-O-C/C-O stretching
1072	1069	1065	1068	1063	1068	1071	
1111	1108	1109	1111	1118	1112	1113	
		1154	1155	1156	1155	1155	
		1203			1203	1205	Amide III
1234	1235		1233				
		1263		1255	1262	1260	
1316	1314	1306		1325	1306	1309	
	1380	1374	1376		1375	1376	CH_x deformation
				1398		1415	
1447	1445	1429	1447		1430	1430	
1519	1515	1550	1522	1588	1554	1554	Amide II
1634	1639		1638		1631	1622	Amide I
		1642			1653	1654	
2876 (shoulder)	2876 (shoulder)	2875	2876 (shoulder)	2875 (shoulder)	2874	2876	CH_x stretching
2931	2933	2930	2929		2923	2927	
2958				2957	2955	2959	
		3096					N-H stretching
		3121			3114	3103	
3277	3285	3283	3279	3270	3285	3262	
		3409			3436	3432	O-H stretching

Figure S1. ATR FTIR spectra of the purified skeletons of *I. basta* after different treatment steps.

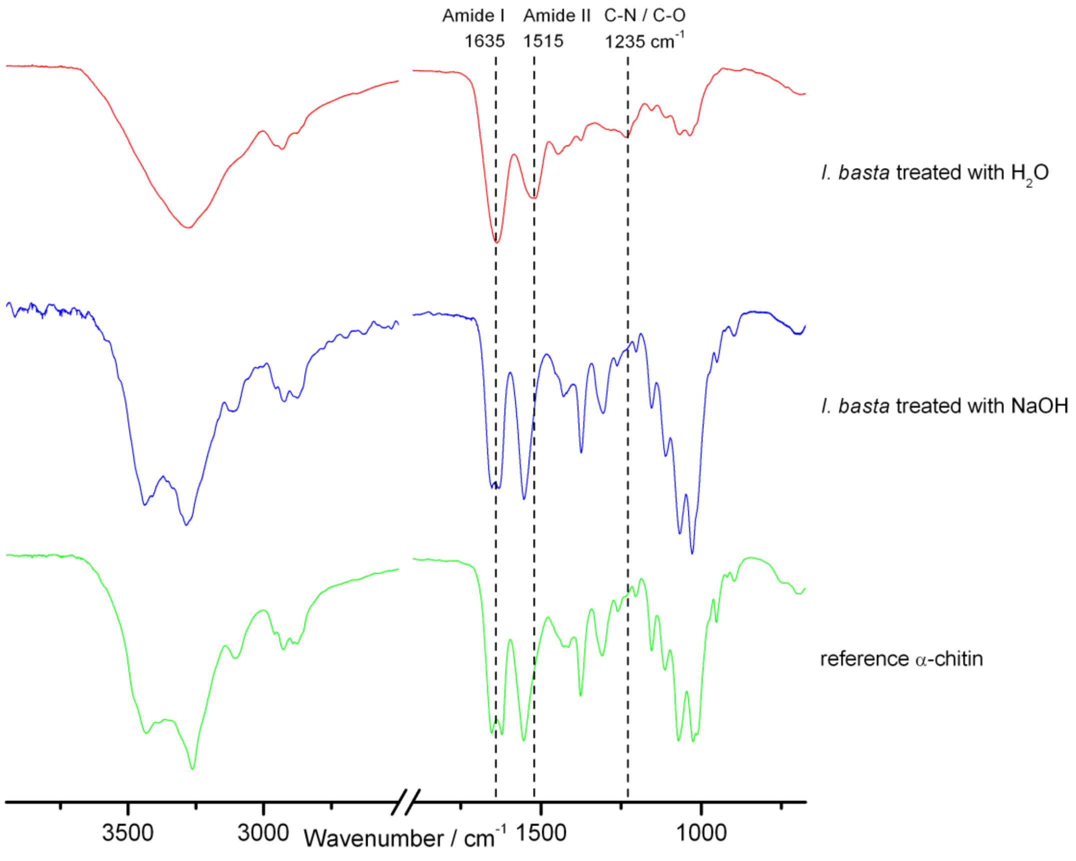

Figure S2. $^{13}C\{^{1}H\}$ CP MAS NMR spectrum and structure of aerothionin as well as assignment table of the observed ^{13}C NMR signals. This symmetric molecule exhibits two pairwise identical carbon positions (e.g., 1 and 1′) which exhibit identical chemical shifts in the liquid-state NMR spectra [1]. The observation of two signals in the solid-state NMR spectrum indicates the presence of two crystallographically different positions for solid aerothionin. * denotes spinning sidebands.

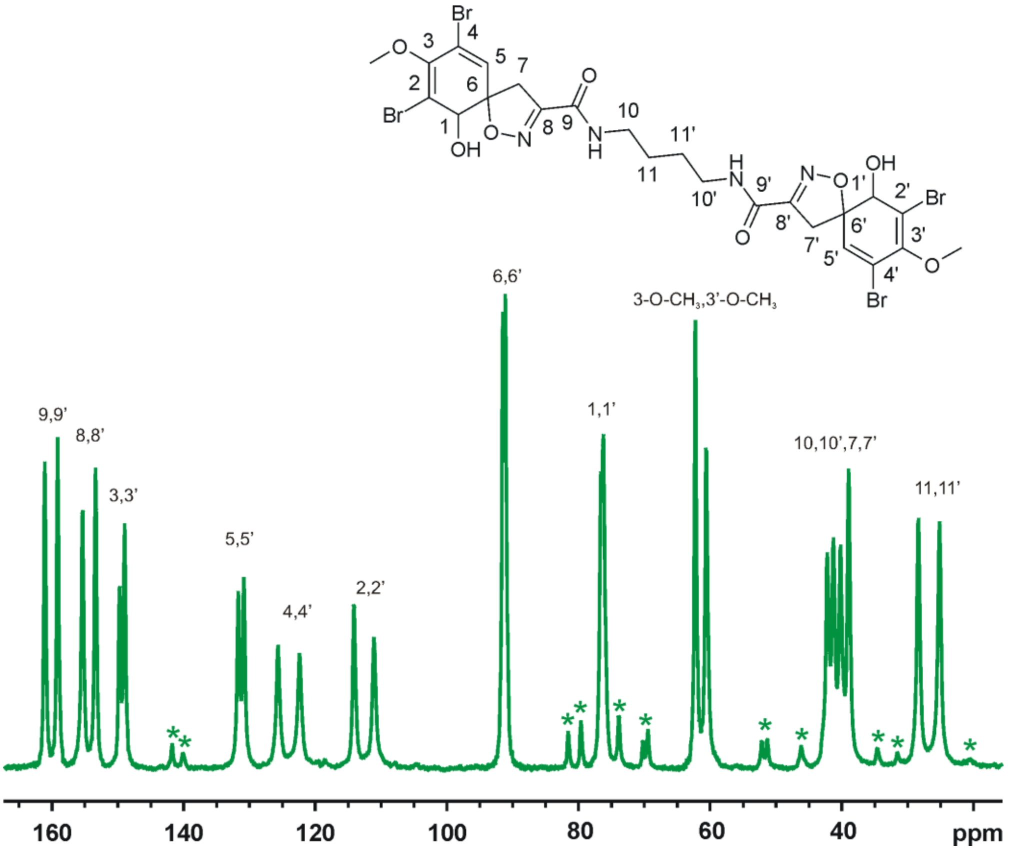

^{13}C NMR signal/ppm	Assignment
25; 28	11/11′
39; 40; 41; 42	7/7′/10/10′
61; 62	3-*O*-CH_3/3′-*O*-CH_3
76; 77	1/1′
91; 92	6/6′
111; 114	2/2′
122; 126	4/4′
131; 132	5/5′
149; 150	3/3′
153; 155	8/8′
159; 161	9/9′

Figure S3. $^{13}C\{^{1}H\}$ CP MAS NMR spectra of the skeletons of *I. basta* after TE100 treatment. For comparison, the spectra of the pure chitin-scaffold obtained after NaOH treatment and of synthetic 5,5′-dibromohemibastadin-1 are also shown. For signal assignments see Figure 4 and Figure S4. * denotes spinning sidebands.

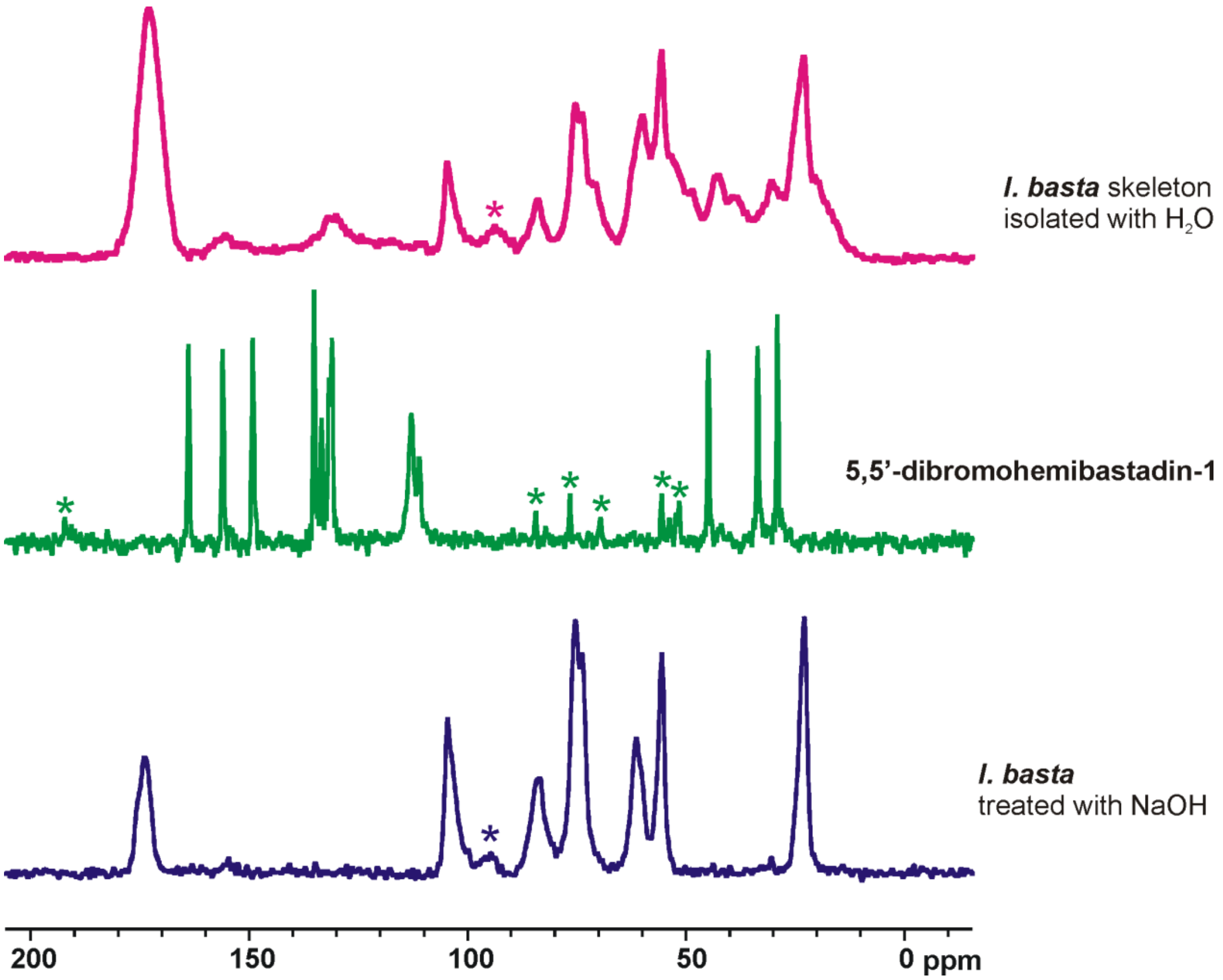

Figure S4. $^{13}C\{^1H\}$ CP MAS NMR spectrum, structure of 5,5′-dibromohemibastadin-1 and assignment table of the ^{13}C NMR signals. * denotes spinning sidebands.

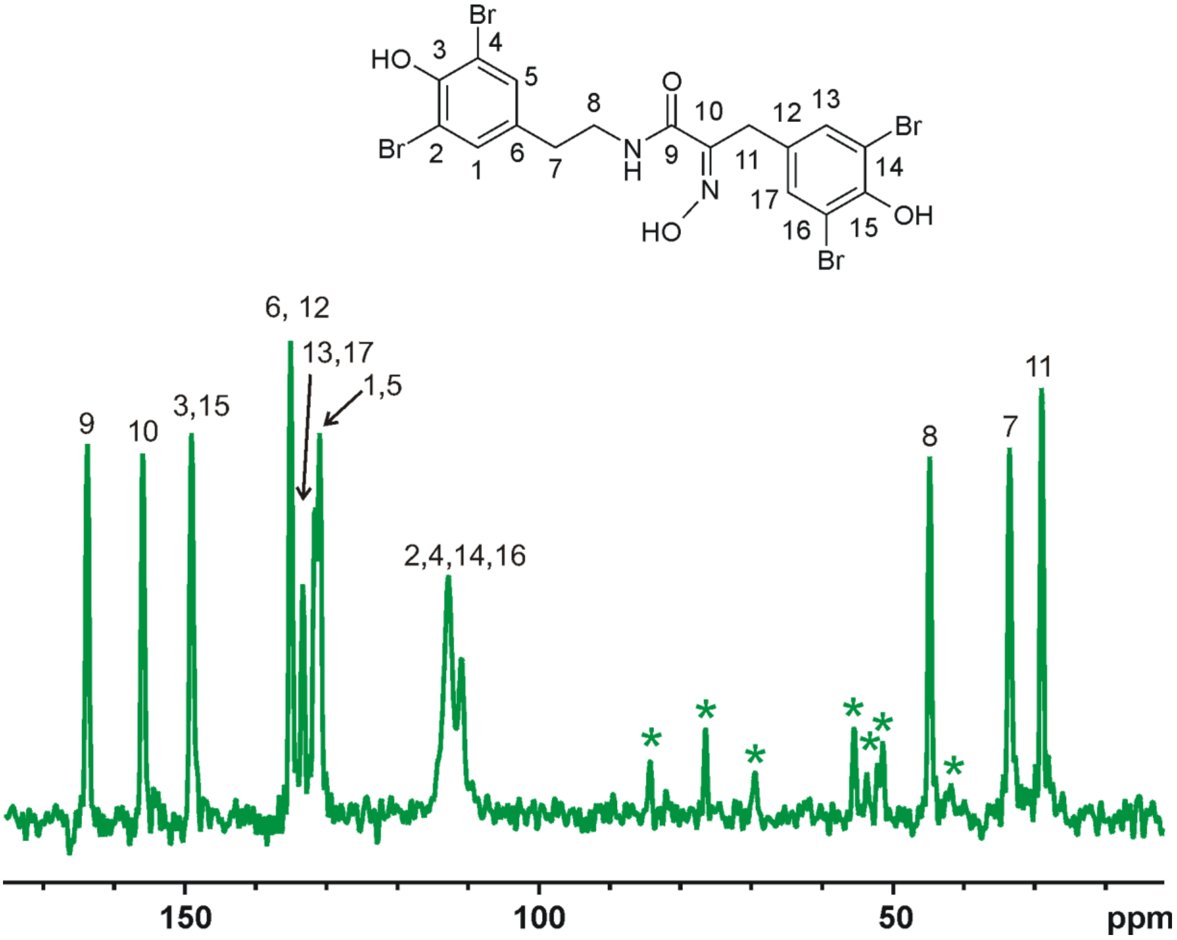

^{13}C NMR signal/ppm	Assignment
29	11
36	7
45	8
111; 113	2/4/14/16
131; 132	1/5
133	13/17
135	6/12
149	3/15
156	10
163	9

Figure S5. ATR FTIR spectra of the purified skeletons of *A. cavernicola* and *I. basta* before and after the MeOH extraction.

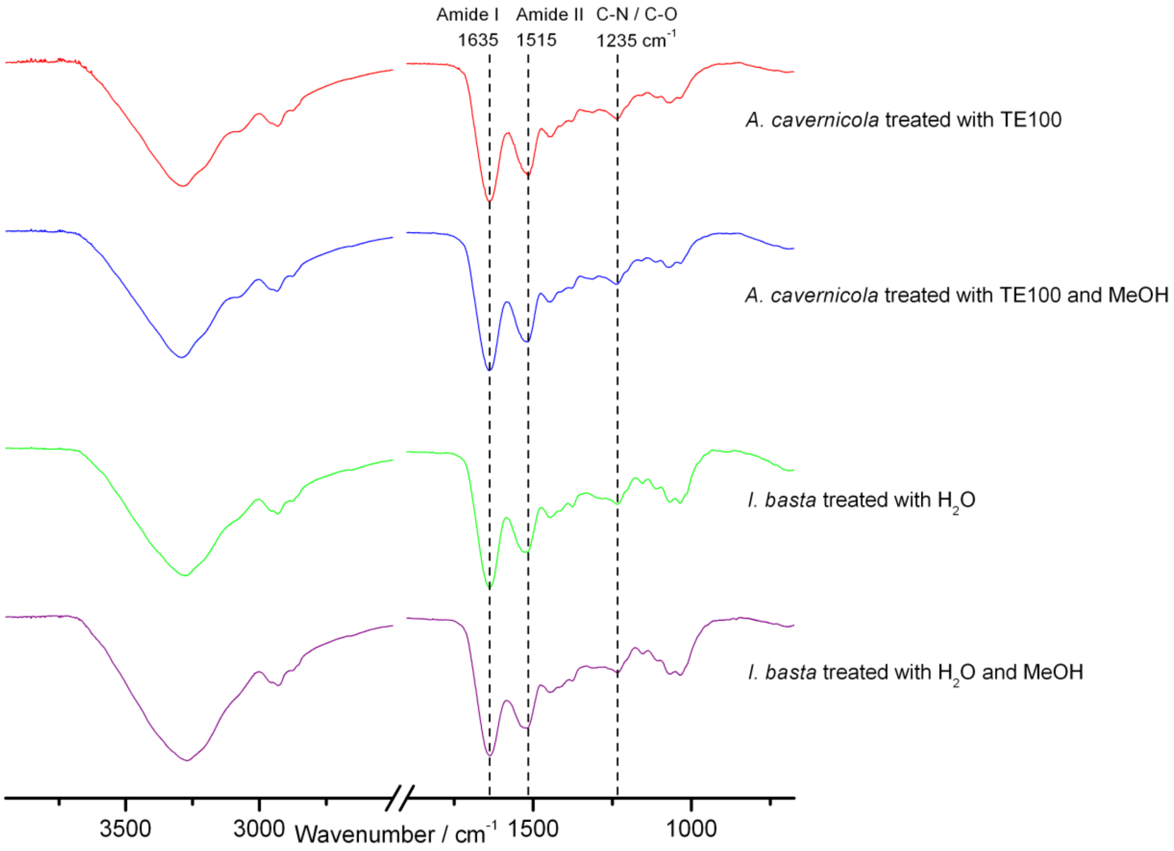

Figure S6. $^{13}C\{^1H\}$ CP MAS NMR spectra of the purified skeletons of *A. cavernicola* and *I. basta* before and after the MeOH extraction.

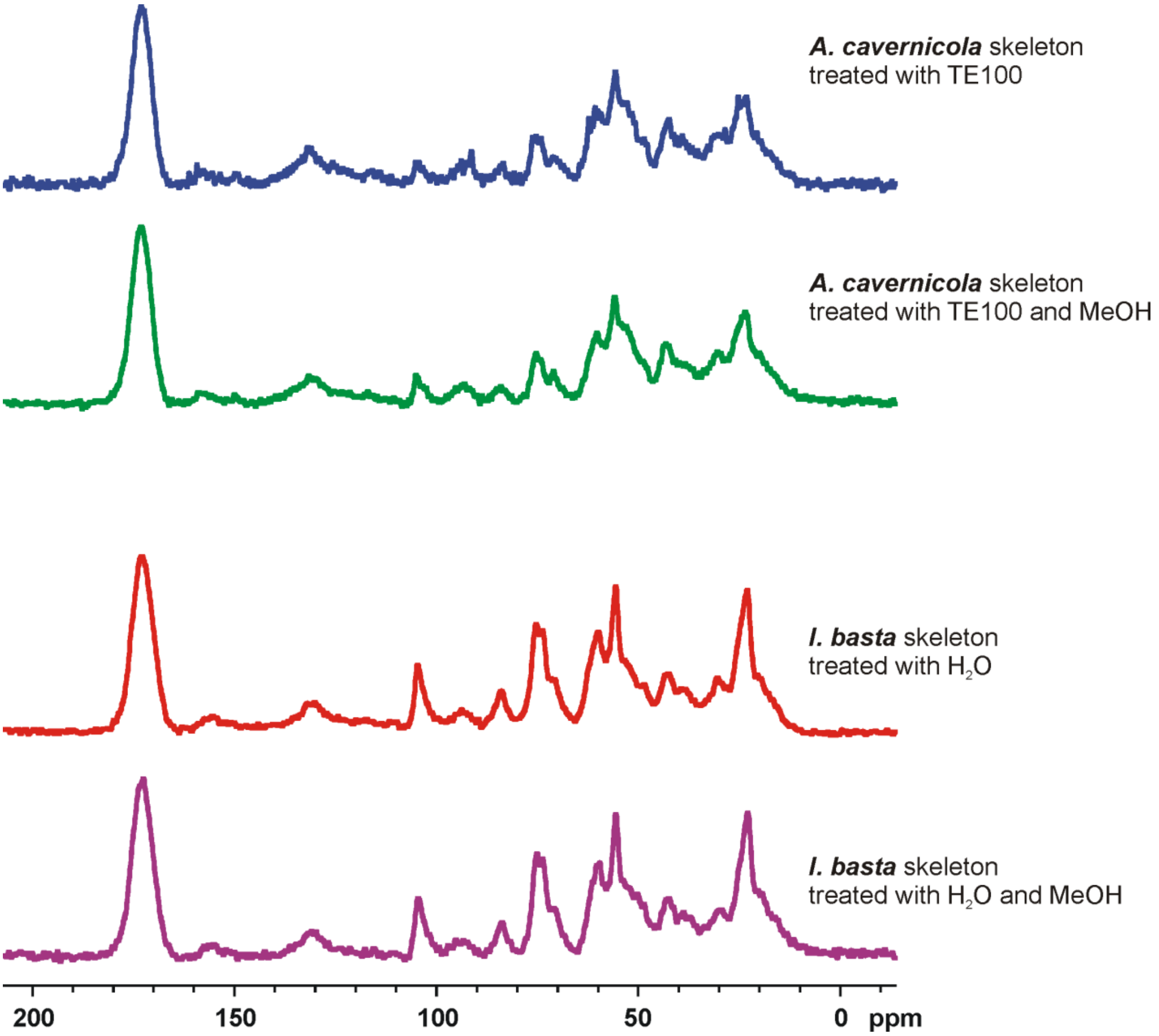

Figure S7. Selected region from the $^{13}C\{^{1}H\}$ CP MAS NMR spectra of the purified skeletons of *A. cavernicola* before and after MeOH extraction and of aerothionin. Note the presence of weak, characteristic signals due to aerothionin before MeOH extraction which disappear after MeOH treatment.

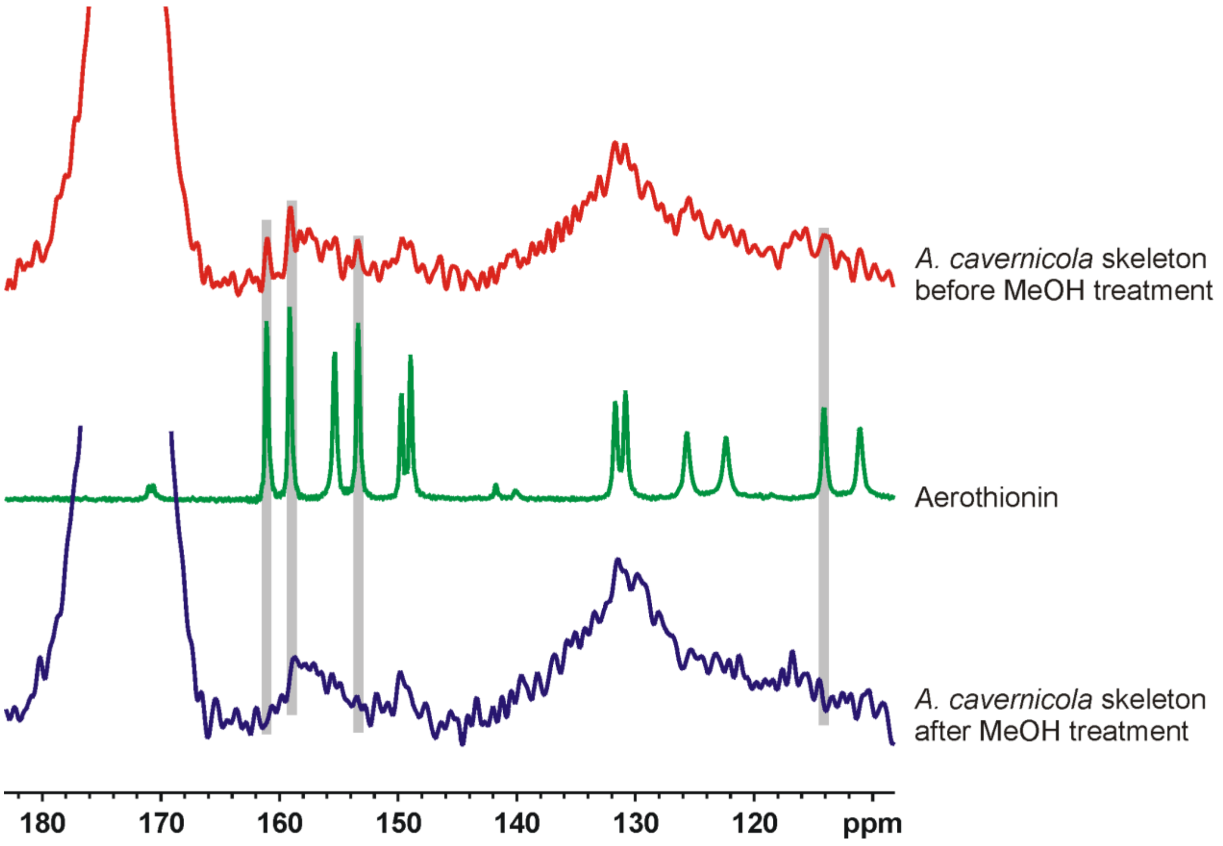

Figure S8. Chemical structures of bromotyrosines identified in the skeleton extracts; (**A**) bastadins 3, 4, 6, 7, 9 and 16 from *I. basta*; (**B**) aerothionin from *A. cavernicola*.

A

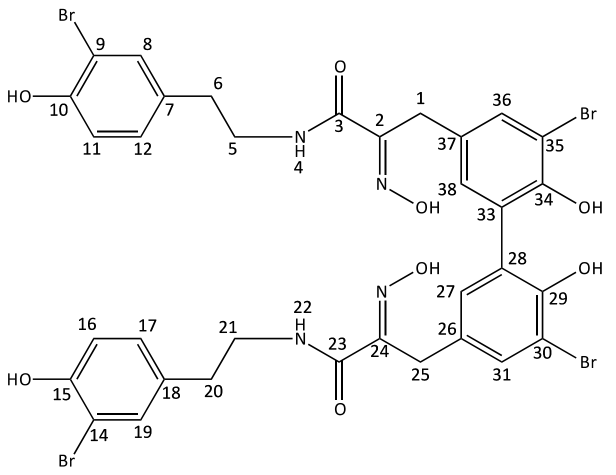

bastadin 3

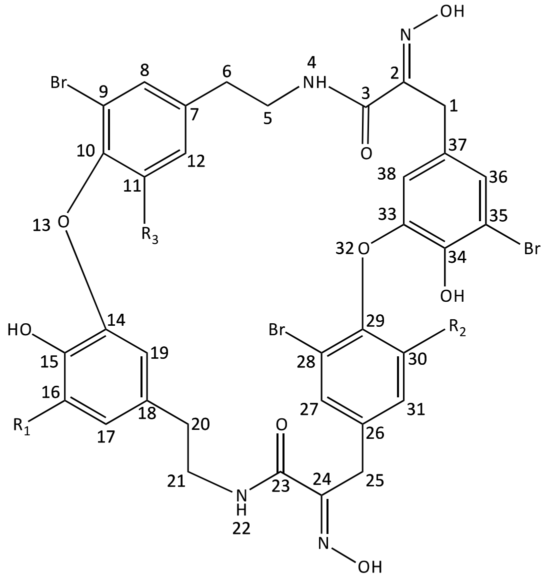

bastadin 4: $R_1 = R_2 = Br$, $R_3 = H$, $\Delta^{5,6}$
bastadin 6: $R_1 = R_2 = R_3 = Br$
bastadin 7: $R_1 = Br$, $R_2 = R_3 = H$, $\Delta^{5,6}$
bastadin 9: $R_1 = R_3 = H$, $R_2 = Br$
bastadin 16: $R_1 = H$, $R_2 = R_3 = Br$

B

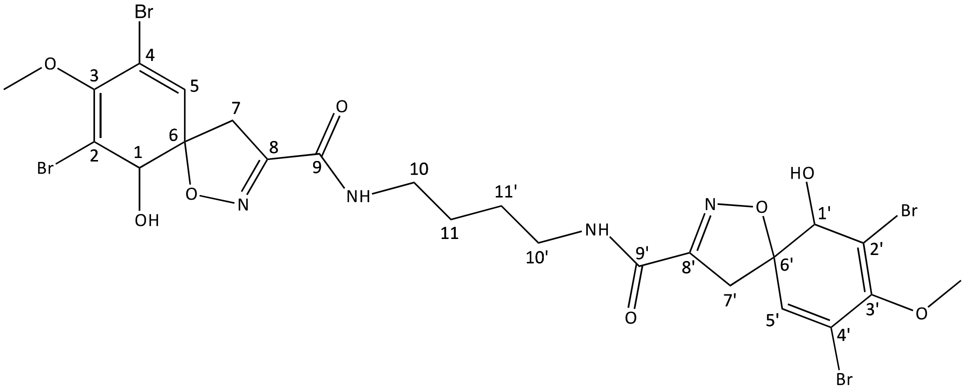

aerothionin

Figure S9. (**A**) Direct injection-ESI-mass spectrum of pure bastadin 3 standard obtained from *I. basta* tissue extract; (**B**) LC-ESI-mass spectrum obtained from a constituent of the *I. basta* skeleton extract. Based on this mass spectrum, this compound was identified as bastadin 3.

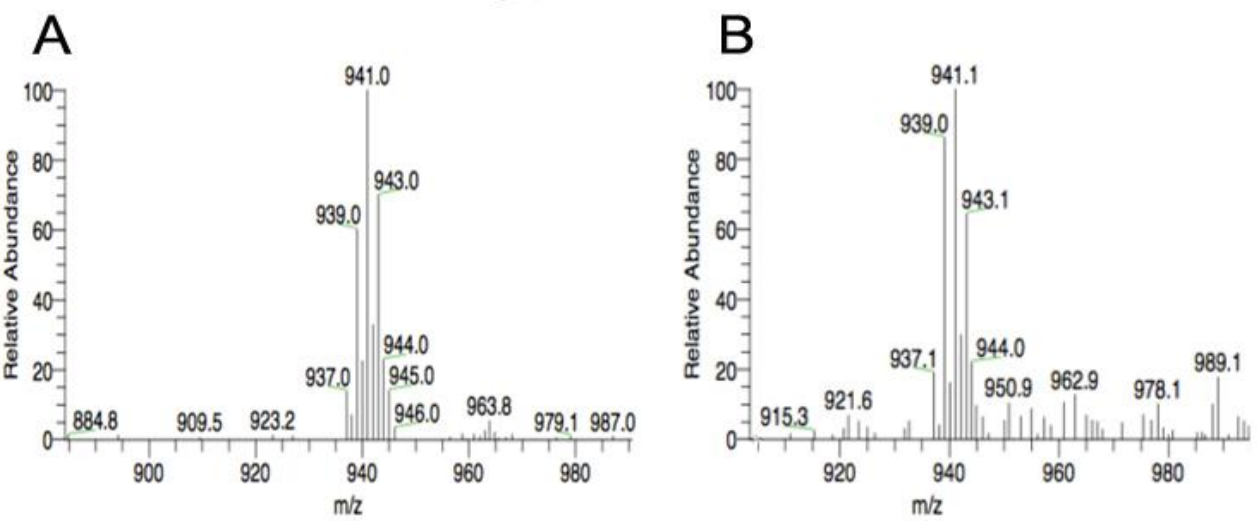

Figure S10. Removal of contaminating pigments (2) from the DNA (1) isolated from bacteria associated with *A. cavernicola*.

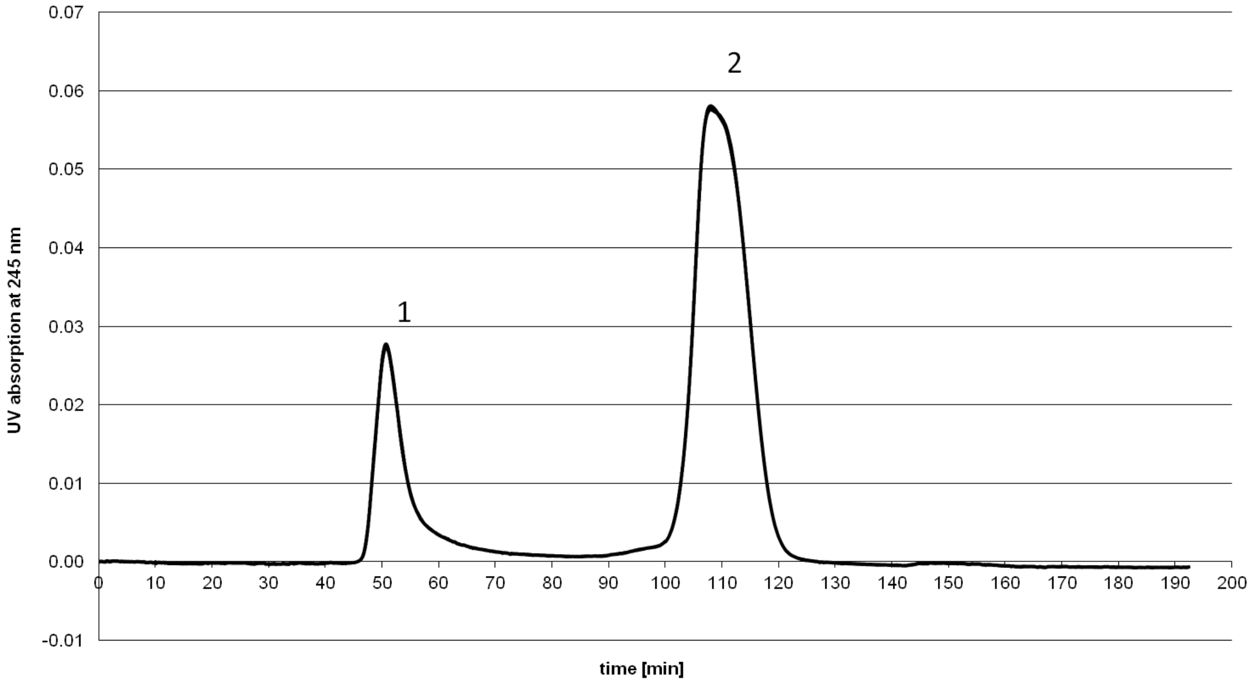

Table S2. Gradient systems for HPLC analysis of *I. basta* and *A. cavernicola*; eluent A: 0.1% formic acid in water, eluent B: MeOH.

I. basta			***A. cavernicola***		
**time [min]**	**eluent A [%]**	**eluent B [%]**	**time [min]**	**eluent A [%]**	**eluent B [%]**
0	60	40	0	90	10
5	60	40	5	90	10
34	25	75	35	0	100
35	0	100	45	0	100
50	90	10	50	90	10
60	90	10	60	90	10

Figure S11. Calibration graph of bastadin 3 for external standard quantification of bastadin derivatives in MeOH-extracts of sponge tissue and skeleton.

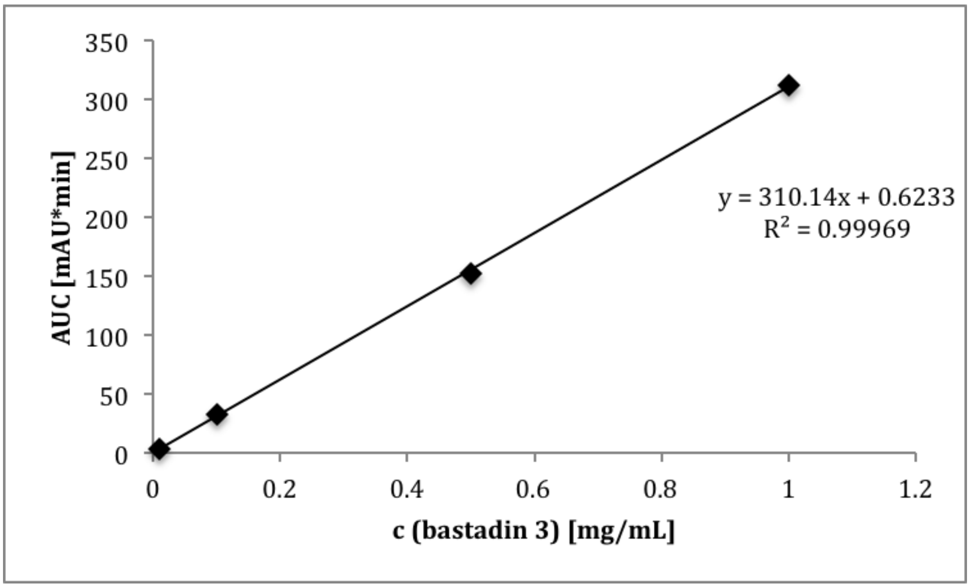

Figure S12. Potentiometric titration curves for quantitative bromine determination in *A. cavernicola* skeleton samples before and after MeOH-extraction. The step in the titration curve represents the bromide ions. The bromine concentration can be determined from the $AgNO_3$ concentration at the inflection point of this potential step.

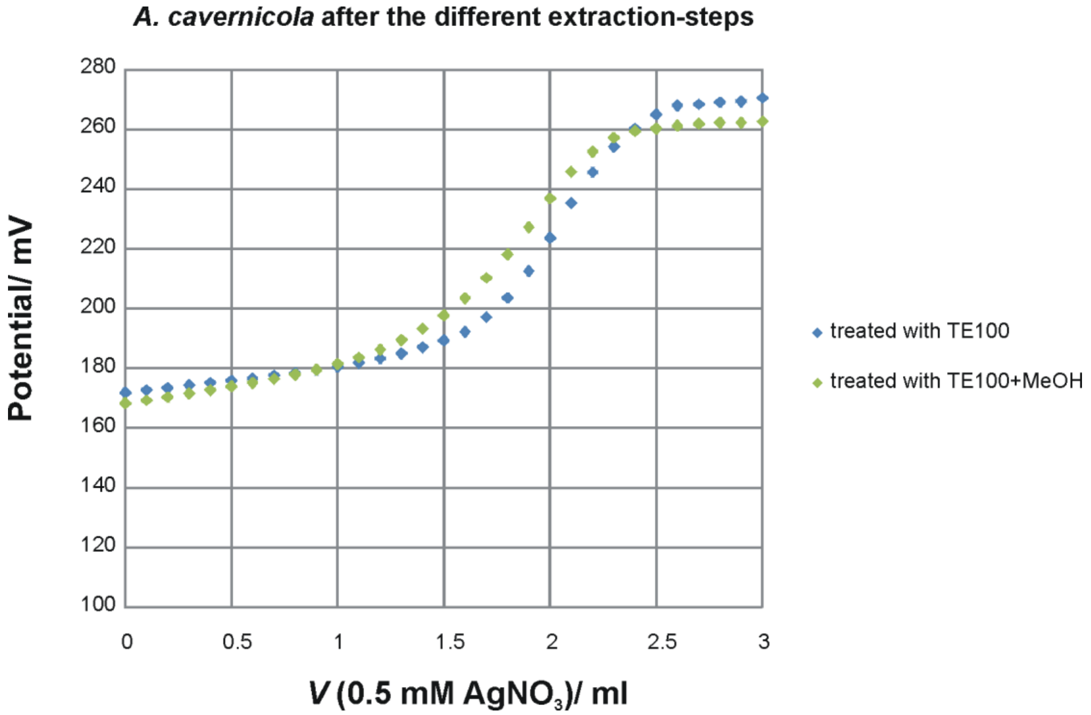

References

1. Kalaitzis, J.A.; Davis, R.A.; Quinn, R.J. Unequivocal ^{13}C NMR assignment of cyclohexadienyl rings in bromotyrosine-derived metabolites from marine sponges. *Magn. Reson. Chem.* **2012**, *50*, 749–754.

© 2013 by the authors; licensee MDPI, Basel, Switzerland. This article is an open access article distributed under the terms and conditions of the Creative Commons Attribution license (http://creativecommons.org/licenses/by/3.0/).
